# Bureau of Health

**Published:** 1886-02

**Authors:** 


Bureau of Health.—The Senator from the Falls River district, in Massachusetts, has introduced a bill to prevent the introduction of contagious and infectious diseases into the United States, and to that end, to create a Bureau of Health, under the auspices of the Department of the Interior. There is to be a "commissioner" chosen from civil life, who is to receive a salary of $4,500. The government is to detail army medical officers to serve as inspectors when and where necessary, or if the "exigencies of the service" require it, civilians may be employed, etc. This is intended as a substitute for the National Board of Health. In our opinion this is a sensible move. Why should there not be a "Commissioner of Public Health," as well as of Agriculture or of anything else? Is not the preservation of the public health a paramount consideration, upon which *all else* depends? Success to the bill!

---

The committee on awards of the American Institute Fair, New York, have awarded the Medal of Superiority to the Jerome Kidder Manufacturing Company, 820 Broadway, New York, for their 1885 exhibit of Electro Medical Apparatuses. For thirteen years the Dr. Jerome Kidder Machines have received the highest awards from the American Institute over all competitors, and wherever they have exhibited in competition.

